# A Case of Idiopathic Tetanus, Treated Successfully by Strychnine

**Published:** 1847-04

**Authors:** Edward Vanderpool


*A Case of Idiopathic Tetanus, treated successfully by Strychnine.* By Edward Vanderpool, M. D. (New York Jour. of Med. Jan., 1847.—Was requested to see Mr. B., aged 58 years, segar merchant. Just one month ago he thought he took cold from sitting in front of his store in the evenings, from a chilliness creeping up the sides of his face, which continued for several days. This was followed by a sore feeling of the face, near the ears, and inability to extend the jaws, with a difficulty of swallowing. About this time, he commenced to fall backwards when walking, which he would do every few days from a sudden spasmodic opisthotonos attack. Two days ago he fell suddenly, in the same way, through the lower half of a window, breaking out both the sash and the glass, but without injury to himself.

I found him with tetanic expression of countenance, permanent rigidity of the masseter and temporal muscles, jaws nearly closed—could only show the edge of the tongue at its tip, which was much bitten, by being caught between his teeth whilst dozing—is frequently drawn, or, if standing, thrown backwards by spasmodic action of the muscles of the back. Bowels soluble. Pulse eighty, and soft. Has taken several cathartic doses of calomel since he felt unwell, and "a number of sweats." Prescribed strychnine in solution, the sixteenth of a grain every two hours.

14th October.—Doctor Fell happening to call on me, I invited him to see the patient. 10 o'clock, A. M.—Took only three doses yesterday and one this morning. Found him with a handkerchief tied around his waist from bad feeling in the region of the diaphragm—produced, as he supposed, by a fall an hour before—rigidity of the jaws about the same; his teeth are worn off, to appearance one-eighth of an inch—thus allowing for the distance he can open his mouth. Spasms more frequent; takes fluid nourishment well. Bowels have moved. ℞. Stryc., one-fourteenth of a grain every two hours. 7 o'clock, P. M.—Jaws have yielded a little, but spasms have been very frequent. ℞. Stryc., one-twelfth of a grain every two hours.

(This treatment was continued with a gradual abatement of the symptoms until the 20th, when the patient was convalescent. He was discharged cured on the 22d.)

Within the last two years, two other cases have occurred in my practice. The lamented young S. Fox, aged 19 years, from an injury whilst bathing, died the fifth day; having been treated with tart ant., assafœt., laudanum and brandy. The other, a coloured woman, two months subsequent to young Fox, recovered. Both were traumatic tetanus. The woman had the opisthotonos form, permanent rigidity, with occasional spasmodic aggravations—especially of the diaphragm—which caused excruciating suffering. The fifth day, her jaws having become firmly closed, I concluded to take the responsibility and bleed her to the extent of a large wash-basin full, when her jaws yielded a little—say $\frac{3}{8}$ths of an inch—and this state of things I endeavored to maintain by tart. ant. She took 4 grs. in solution every hour and every other hour for two days without producing emesis, when, for fear of inflammation of the stomach, I suspended its use. Her convalescence was very slow, but her health has been good since.

*N. Y., Oct. 10th*, 1846.

P. S. Strychnine will not dissolve in water or alcohol. A good way is to add ʒj. acetic acid to ʒvij. aq., which will make a clear solution.—*Ibid.*

